# A Novel Method of Frequency Band Selection for Squared Envelope Analysis for Fault Diagnosing of Rolling Element Bearings in a Locomotive Powertrain

**DOI:** 10.3390/s18124344

**Published:** 2018-12-09

**Authors:** Lang Xu, Steven Chatterton, Paolo Pennacchi

**Affiliations:** Department of Mechanical Engineering, Politecnico di Milano, Via G. La Masa 1, 20156 Milan, Italy; steven.chatterton@polimi.it (S.C.); paolo.pennacchi@polimi.it (P.P.)

**Keywords:** roller element bearing diagnostics, squared envelope analysis, frequency band selection, locomotive powertrain

## Abstract

The development of diagnostics for rolling element bearings (REBs) in recent years makes it possible to detect faults in bearings in real-time. Squared envelope analysis (SEA), in which the statistical characteristics of the squared envelope (SE) or the squared envelope spectrum (SES) are analysed, is widely recognized as both an effective and the simplest method. The most critical step of SEA is to find an optimal frequency band that contains the maximum defect information. The most commonly used approaches for selecting the optimal frequency band are derived from measuring the kurtosis of the SE or the SES. However, most methods fail to cope with the interference of a single or a few impulses in the corresponding domain. A new method is proposed in this paper called “PMFSgram”, which just calculates the kurtosis of the SES in the range centred at the first two harmonics with a span of three times the modulation frequency rather than the entire SES of the band filtered signals. It is possible to avoid most of the interference from a small number of undesired impulses in the SES. PMFSgram uses several bandwidths from 1.5 times to 4.5 times the fault frequency and for each bandwidth has the same number of central frequencies. The frequency setting method is able to select an optimal frequency band containing most of the useful information with less noise. The performance of the new method is verified using synthesized signals and actual vibration data.

## 1. Introduction

Condition monitoring and health management are important for the safe operation of modern locomotives. An REB is one of the most important components in a locomotive powertrain and is prone to failure. Vibration-based REB fault diagnosis is established based on the fundamental fact that an early stage defect of the bearing would generate a series of repetitive impacts [1]. Signals of REB with incipient defects are cyclostationary, because of the existence of slip between the rolling elements and rings [2]. The SES has become the most widely used technique for REB fault diagnosis due to its simplicity and strength in dealing with cyclostationary signals [3,4]. A critical step before carrying out the SES is the selection of an optimal frequency band that contains the most information about the bearing fault. In the last two decades, much work has been done on optimal frequency band selection, and several useful methods have been developed. The formal spectral kurtosis (SK) method has been proposed for the first time and has demonstrated its ability to detect the existence of a series of transients and to point out their location in the frequency domain [5,6,7]. Calculating the SK of the entire SES is time consuming and not advisable for a real time application; thus, the fast kurtogram method with a fast algorithm was proposed based on an arborescent multirate filter-bank structure and STFT demodulation (or FIR demodulation) [8]. The fast kurtogram method calculates the kurtosis of the SE in every frequency band divided by the algorithm. The frequency band with the maximum value of kurtosis is seen as a potential candidate for the optimal frequency band. Despite the fact that the fast kurtogram can be used to select the optimal frequency band for REB fault diagnosis in some cases, the performance of the fast kurtogram was limited due to its shortcomings, such as sensitivity to a single impulse in the signal and insensitivity to high repetition rate impulses in the signal. Later minimum entropy deconvolution was used to enhance the performance of SK in rolling bearing fault diagnosis [9]. The protugram method was proposed to measure the kurtosis of the SES instead of the SE of the band filtered signal [10]. Even though the effectiveness and merits of the protugram method have been demonstrated in many cases, it is still impossible to overcome the shortcomings, similar to those of the fast kurtogram mentioned above in the frequency domain. The bandwidth of the protugram method is also fixed at approximately three times the fault frequency, which may miss useful information when the bandwidth is too small compared to the sampling frequency or include too much useless information when the bandwidth is too large. An improved kurtogram method using a wavelet packet transform (WPT) to filter the signal was proposed by Lei [11]. They proved that WPT can be used to improve the process of filtering compared with STFT demodulation (or FIR demodulation). However, the essence of the method still requires an evaluation of the kurtosis of the SE; thus, it inherits the shortcomings of the fast kurtogram. The enhanced kurtogram evaluates the kurtosis of the power spectrum of the envelope of the band filtered signal, which is filtered by WPT [12]. The performance of this method is improved in comparison to the performance of the fast kurtogram, but it also faces the disadvantage of being too sensitive to interferential single or few impulses in the power spectrum. Moreover, the filter banks of WPT are based on a binary tree, which would cause frequency leakage when the resonance frequency of the fault signal is near a quarter of the sampling frequency [11]. The PeaksMap is introduced to evaluate the peak index based on the existence of cyclic components in the SES [13]. A set of central frequencies and frequency bandwidths are defined in advance. The corresponding statistical spectral threshold of the SES of the signal in each frequency band needs to be calculated based on non-white noise [14,15]. Its performance has been proven by much actual vibration data. The infogram method uses negentropy to measure the degree of instability of the signal [16]. It combines the negentropy of the SE and the SES of the band filtered signals, but using this method is not convenient for an automatic algorithm. A method for enhancing the fault diagnosis of a rotating machine was proposed in [17,18]. Song developed a signal feature extraction and fault diagnosis method for bearing diagnosis of low-speed machinery [19]. A three-dimensional geometric features-based sparse component method for compound faults diagnosis was proposed by Hao [20]. A maximum correlated kurtosis deconvolution method was proposed to extract the fault frequency component from the collected vibration signal [21]. Compared with the aforementioned methods, the key step and main advantage of the new method is that only the SES centred at the first two harmonics with a span of three times the modulation frequency is used to calculate the kurtosis. Thus, the assumption that the repetitive impacts excited by a local failure correspond to peak values at the fault frequency and its multiples in SES can be used optimally. The situation in which a selected frequency band includes a single or few impulses in the SES with high kurtosis value can also be avoided, if these impulses are not in the range used by the PMFSgram.

The paper is organized as follow: Section 2 reviews the SK method. Section 3 depicts the algorithm of the new method in detail. Section 4 verifies the performance of the new method using synthetic signals. Section 5 demonstrates the performance of the new method with actual vibration data.

## 2. Review of the SK Method

Kurtosis is a statistical index which can be used to measure the peakedness or flatness of a signal [22]. While the formal definition of the SK is proposed by J. Antoni using the Wold–Cramér decomposition with the assumption of seeming the signal as a conditionally non-stationary process [6]. It can be used to measure the peakedness of the SE at a certain frequency f [6]. For a continuous time-signal, the definition of the SK is

(1) $$K(f)=\frac{S_{4}(f)}{S_{2}^{2}(f)}-2$$

where $S_{4}(f)$ and $S_{2}(f)$ represent the averaged fourth and second instantaneous moment at the frequency f, respectively. For a real signal, subtract 3 instead of 2.

Calculating the SK of the entire SES is uneconomical for real-time use; thus, the fast kurtogram is proposed [8]. The kurtosis of the complex envelope of the signal at each node is displayed in the corresponding block in the fast kurtogram, and the calculation of the kurtosis for each node is [8]

(2) $$K=\frac{\langle |c[n]{|}^{4}\rangle }{{\langle |c[n]{|}^{2}\rangle }^{2}}-2$$

where c[n] is the complex envelope of the band filtered signal at one node and 〈…〉 represents the averaging operation.

The SES of the band filtered signal in the node with the maximum kurtosis will be used to recognize whether there are fault frequencies that represent a local defect in the bearing. The fast kurtogram is visualized, and the algorithm is convenient for automatic operation.

## 3. The Algorithm of the New Method

The fault signal of an REB is a cyclostationary signal that can be detected by measuring the kurtosis of the SE in the time domain [8], and it can also be detected by measuring the kurtosis of the SES in the frequency domain [9]. Moreover, it is more effective in the frequency domain when the signal-to-noise ratio (SNR) is low [9]. Different bearing fault types have corresponding characteristic fault frequencies which are determined by the shaft rotation frequency and structure parameters. Fault frequencies for defects on the outer ring (BPFO) and roller (BSF) [23] are as follows:

(3) $$\mathrm{BPFO}=\frac{N_{b}}{2}f_{r}(1-\frac{D_{b}}{D_{p}}\cos\alpha )$$

(4) $$\mathrm{BSF}=\frac{D_{p}}{D_{b}}f_{r}[1-{\left(\frac{D_{b}}{D_{p}}\cos\alpha \right)}^{2}]$$

where $N_{b}$ is the number of the roller element, $f_{r}$ is the shaft rotation frequency, $D_{b}$ and $D_{p}$ are roller diameter and pitch diameter respectively, α is the contact angle.

**Figure 1:**
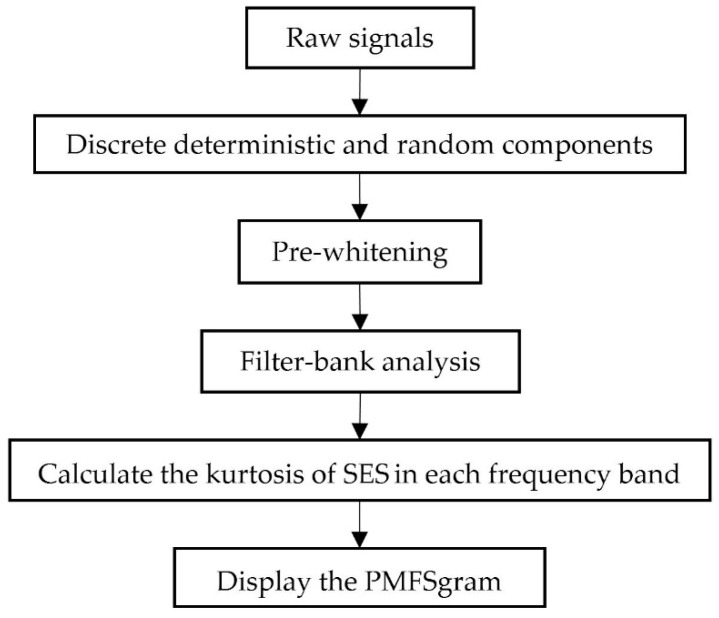
The flowchart of the new method.

In the new method, only part of the SES of the band filtered signal is used to calculate the kurtosis. The flowchart of the new method is displayed in Figure 1, and the steps of the algorithm are as follow:

Step1: Discretize the deterministic and random components of the signal. Generally, in a real situation, the collected vibration signal is a compound signal containing several components of the REBs, the rotation shafts, the gears and other rotational parts [1]. The bearing signals exhibit some randomness and are seen as cyclostationary, while the shafts and gear signals are treated as purely periodic. The signal of a bearing with an incipient defect is very weak and easily overwhelmed by gear signals and shaft signals. Therefore, this step is very important for obtaining strong peak values at the fault frequency or multiples in the SES, which indicate the existence of a bearing fault. Applied methods for separate deterministic and random components are reviewed in ref. [24]. Computed order tracking (COT) and time synchronous averaging (TSA) are used here [25,26].

Step2: Signal pre-whitening. As it is impossible to eliminate the deterministic components in the signal completely in step 1, an additional pre-whitening step can be used to further unmask the weak bearing signals [27]. Cepstrum analysis and linear prediction are the most commonly used approaches [24], and linear prediction is used here. Linear prediction uses several points in the near past to predict the deterministic component of a signal. The difference between the signal x(n) and its deterministic component is the unpredictable part e(n). Thus,

(5) $$x(n)=-\sum_{k=1}^{p}a(k)x(n-k)+e(n)$$

where the first term on the right is the deterministic part, p is the order of the model, vector a is the coefficient vector of the autoregressive model.

Using linear prediction, the deterministic components are removed, leaving the pre-whitened residual part which contains the bearing signal. Often a small order is used in bearing fault diagnosis. More details about linear prediction can refer to reference [24]. Pre-whitening is not mandatory but is recommended especially when the kurtosis of the signal after step 1 is low.

**Figure 2:**
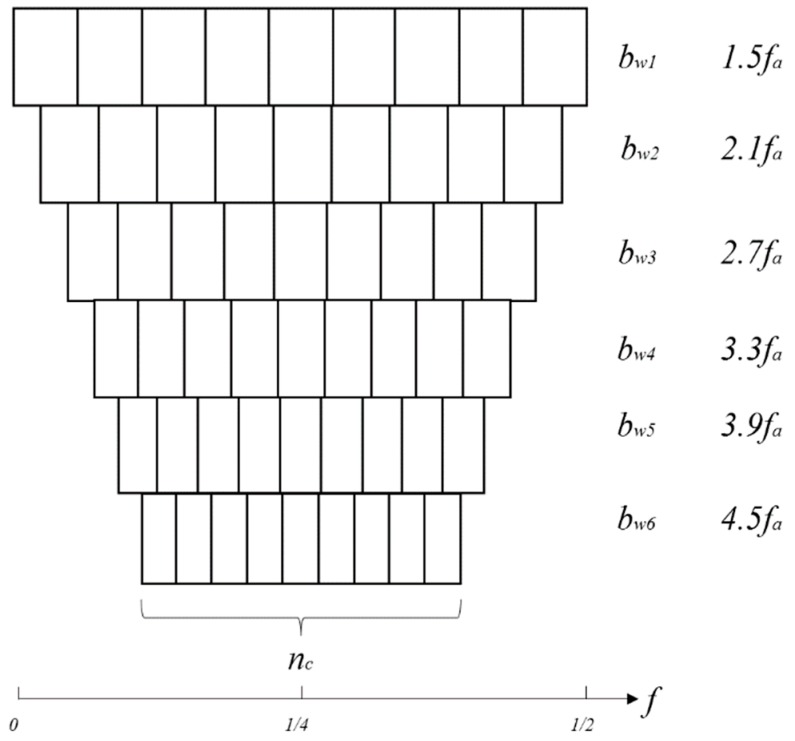
Schematic diagram of the filter-bank analysis.

Step3: Filter-bank analysis. A schematic diagram of the filter-bank analysis is displayed in Figure 2. FIR filter is used here because of its convenience and ease of implementation. Similar to the PeaksMap a set of frequency bandwidths is selected in advance. The frequency bandwidth varies from 1.5 times to 4.5 times the fault frequency *f_a_* and the increment between the two bandwidths is 0.6 times the fault frequency *f_a_*. The following two suggestions are made: the minimum bandwidth should be larger than four times the modulation frequency *f_r_* (the rotational frequency of the shaft); otherwise, there is less useful information in the band filtered signal and the maximum bandwidth should be smaller than half of the Nyquist frequency; it is useless having a bandwidth greater than half of the Nyquist frequency. The total number of the central frequencies for every bandwidth is a constant. The central frequencies of each bandwidth form an arithmetic progression. The central frequency step size for each bandwidth is calculated as:

(6) $$\Delta f_{c}=\frac{F_{s}/2-b_{w}}{n_{c}}$$

where $b_{w}$ is one of the frequency bandwidths, $\Delta f_{c}$ is the central frequency step size corresponding to a certain $b_{w}$, $F_{s}$ is the sampling frequency, $n_{c}$ is the total number of the central frequencies for each bandwidth. The selection of the constant $n_{c}$ should ensure that $\Delta f_{c}$ for the minimum bandwidth $b_{w1}$ is smaller than the bandwidth.

**Figure 3:**
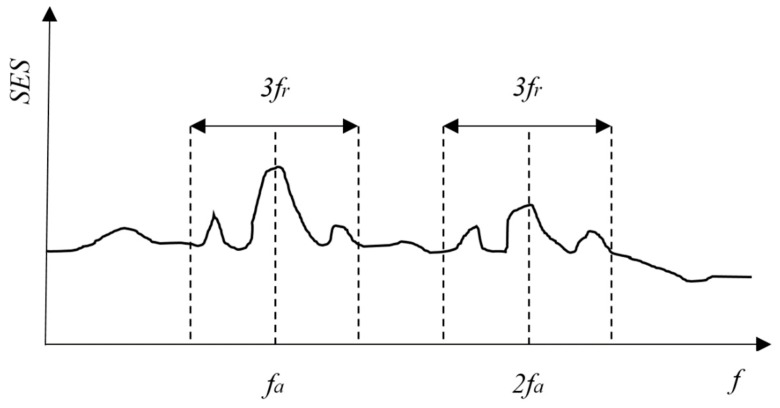
The part of squared envelope spectrum (SES) used to calculate the kurtosis.

Step4: Calculate the kurtosis of the SES in each frequency band. This is the key step of the algorithm. Not the entire SES but part of it is used to calculate the kurtosis. It is not trivial to detect the third harmonic or higher harmonics in the SES because they may be overwhelmed by the background noise when the SNR of the signal is below 1 [28]. It is also reasonable to say that there is a bearing fault if there are peak values at the fault frequency and the second harmonic in the SES. Therefore, only the SES in the range centred at the fault frequency *fa* and the second harmonic *2fa*, with the span of three times the modulation frequency *f_r_* are used to calculate the kurtosis of the SES (see Figure 3). Since the occurrence of the fault frequency is usually accompanied by sidebands, the span is set to three times the modulation frequency to include the first two sidebands. The kurtosis for one node in Figure 2 with a certain central frequency and bandwidth is given as:

(7) $$K(f_{c},b_{w})=\frac{\langle {\left(|SES_{f_{a}}[n]|-\langle \left|SES_{f_{a}}[n]\right|\rangle \right)}^{4}\rangle }{{\langle {\left(|SES_{f_{a}}[n]|-\langle \left|SES_{f_{a}}[n]\right|\rangle \right)}^{2}\rangle }^{2}}-2$$

where *fc* is the central frequency and $SES_{f_{a}}[n]$ is the squared envelope spectrum selected as in Figure 3.

## 4. Numerical Experiments

This section aims to illustrate the process of the PMFSgram, to test its performance, and then to compare its performance with that of the protugram method, the enhanced kurtogram method, and the PeaksMap. The first experiment tests the performance of the new method when dealing with the high repetition rate of impacts with a high SNR. The second experiment tests the performance of the new method when dealing with a high repetition rate of impacts with a low SNR. The third experiment tests the ability to resist the interference of single or few impulses in the SES. Since only white Gaussian noise (WGN) is added to the synthesized signals, the pre-processing is omitted. Additionally, the total number of central frequencies for every bandwidth of the PMFSgram in these cases is 60.

### 4.1. High Repetition Rate of Impacts with High SNR

**Figure 4:**
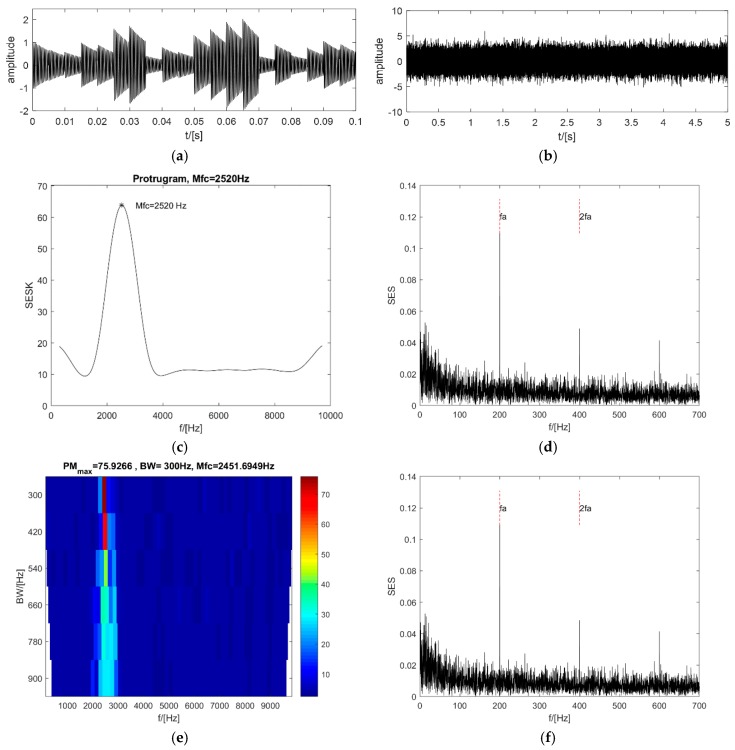

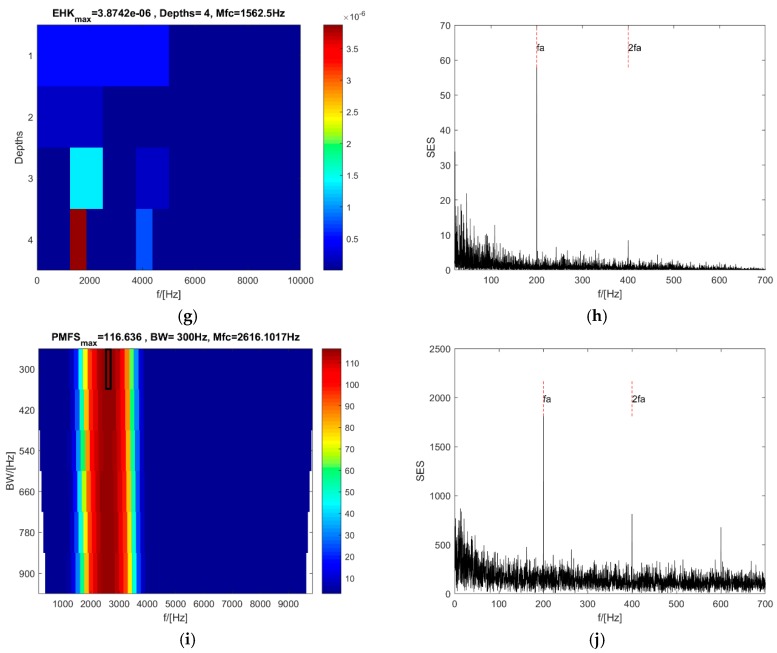
Analysis results of 4.1: (**a**,**b**) the synthesized signals before white Gaussian noise (WGN) noise is added and after WGN noise is added; (**c**,**d**) protugram and the SES of its optimal frequency band; (**e**,**f**) PMgram and the SES of its optimal frequency band; (**g**,**h**) The enhanced kurtogram and the power spectrum of its optimal frequency band; (**i**,**j**) PMFSgram and the SES of its optimal frequency band. * Mfc means the optimal central frequency and fa, 2fa represent the repetitive frequency (fault frequency) and its second harmonic.

A series of impacts with a high repetition rate are generated based on Ref. [16,28]. A single impact is generated by a single degree-of-freedom damping oscillation system with an amplitude of 1, a resonance frequency of 2500 Hz, and a decaying coefficient of −100. The amplitude of the whole signal has a 10% fluctuation, and the period of the signal has a jitter of 5%. The length of the signal is 10^5^ and the sampling frequency is 20,000 Hz. The repetition rate of the impact is 200. A white Gaussian noise with SNR = −6 dB is added to the signals. The corresponding analysis results are shown in Figure 4.

For clarity, only 0.1 s of the synthesized signal without the WGN is shown in Figure 4a. Figure 4b is the entire synthesized signal after the WGN is added. It is clearly shown in Figure 4c that the maximum value in the protugram occurs at the central frequency of 2520 Hz, quite close to the resonance frequency of 2500 Hz. The corresponding SES of the optimal frequency band also clearly shows two peak values at the repetitive frequency of the impacts and the second harmonic (see Figure 4d). For a single set of the signals, the PMgram is used instead of the PeaksMap. The frame of the PMgram is the same as that of the PMFSgram. It can be seen from Figure 4e that the optimal central frequency is 2451.69 Hz and the optimal bandwidth is 300 Hz. The enhanced kurtogram can detect the impacts exactly as well (see Figure 4h), even though the optimal central frequency is 1562.5 Hz and the optimal bandwidth is at the fourth level (see Figure 4g). By contrast, the optimal central frequency of the new method PMFSgram is 2616.1 Hz and the optimal frequency bandwidth is 300 Hz (see Figure 4i). The optimal frequency bandwidth is 1.5 times of the repetitive frequency of the impacts, but not three times. This demonstrates that the bandwidth of three times the fault frequency used in the protugram may contain too much useless information in some cases. The SES of the optimal frequency band is shown in Figure 4j, which clearly displays the repetitive frequency and the second harmonic of the impacts. Therefore, the new method has the ability to detect impacts with a high repetition rate, and the new method can be a potential candidate for detecting the early stages of local failure in an REB.

### 4.2. High Repetition Rate of Impacts with a Low SNR

**Figure 5:**
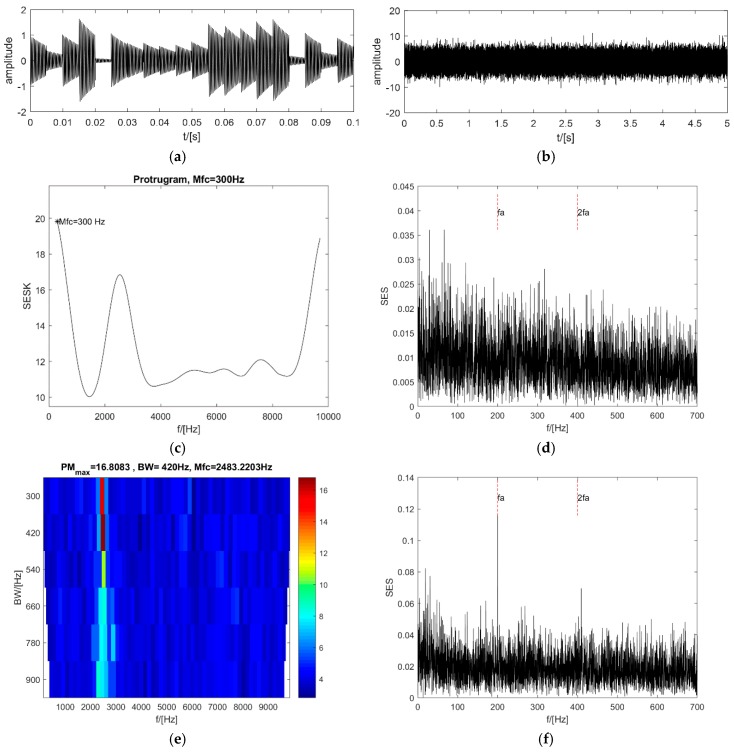

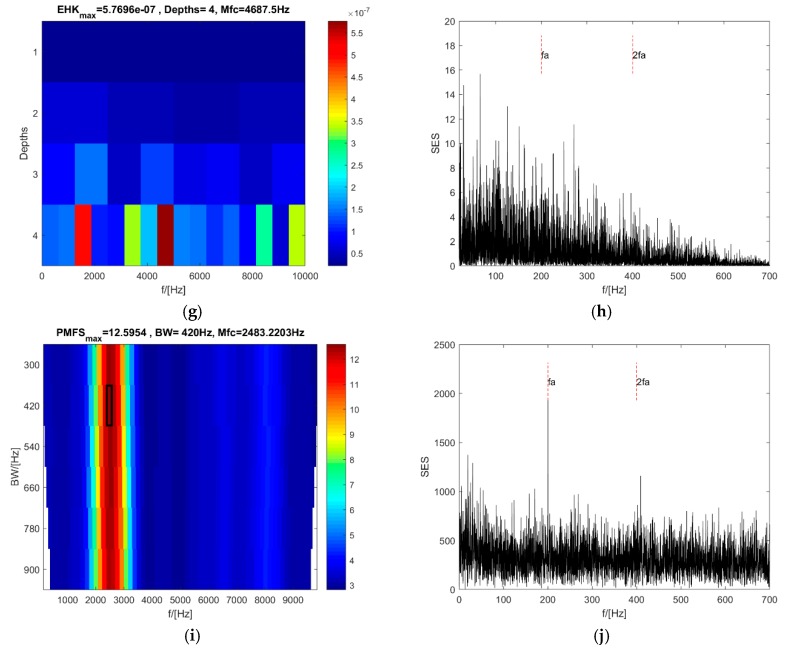
Analysis results of 4.2: (**a**,**b**) the synthesized signals before WGN noise is added and after WGN noise is added; (**c**,**d**) the protugram and the SES of its optimal frequency band; (**e**,**f**) the PMgram and the SES of its optimal frequency band; (**g**,**h**) the enhanced kurtogram and the power spectrum of its optimal frequency band; (**i**,**j**) the PMFSgram and the SES of its optimal frequency band.

When the SNR of the signal in Section 4.1 decreases to −12 dB (see Figure 5a,b), even though there is still a peak value near the resonance frequency in the protugram, the optimal central frequency is selected automatically using the maximum kurtosis of the SES is 300 Hz (see Figure 5c), and the SES of the selected optimal frequency band is unable to point out the existence of the impacts (see Figure 5d). By contrast, the PMgram can still select an optimal central frequency close to the resonance frequency of the impacts at 2483.22 Hz, and the optimal frequency bandwidth is 420 Hz (see Figure 5e). The SES of the optimal frequency band of the PMgram is a bit weaker than in Section 4.1, but it still can indicate the existence of the impacts clearly (see Figure 5f). The enhanced kurtogram is displayed in Figure 5g, in which the optimal central frequency is 4687.5 Hz and the optimal bandwidth is 625 Hz at the fourth level. But in Figure 5h the SES of the optimal frequency band of the enhanced kurtogram has difficulty detecting the impacts. However, it is shown in Figure 5i that the optimal central frequency is 2483.22 Hz and the optimal bandwidth is 420 Hz for the PMFSgram. The optimal bandwidth is 2.1 times of the repetitive frequency of the impacts. The corresponding SES of the optimal frequency band is displayed in Figure 5j and indicates the existence of the impacts clearly. This case has proved that the new method is capable of detecting the impacts even though the noise is very heavy.

### 4.3. Interference of a Few Impulses in the SES

**Figure 6:**
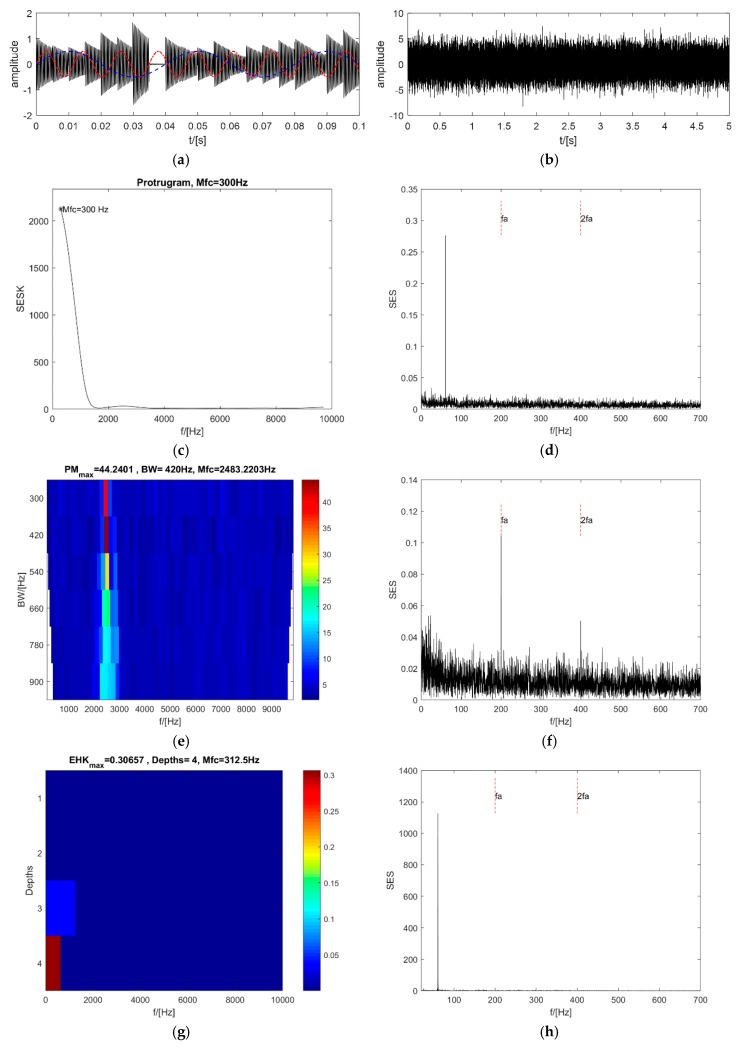

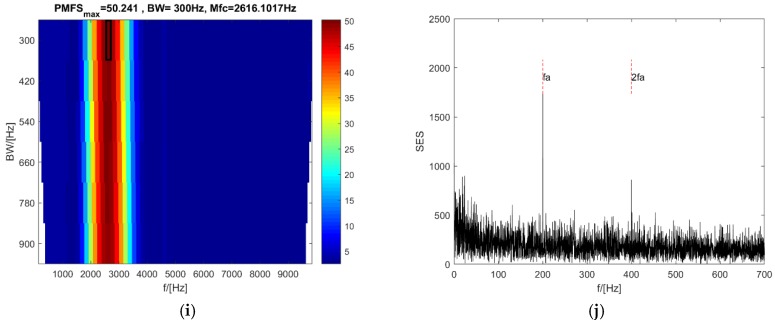
Analysis results of 4.3: (**a**,**b**) the synthesized signals before WGN noise is added and after WGN noise is added; (**c**,**d**) the protugram and the SES of its optimal frequency band; (**e**,**f**) the PMgram and the SES of its optimal frequency band; (**g**,**h**) the enhanced kurtogram and the power spectrum of its optimal frequency band; (**i**,**j**) the PMFSgram and the SES of its optimal frequency band.

The foundation of the synthesized signal is the same as in Section 4.1, except that two pure sinusoidal signals are added as the interferential impulses in the SES (see Figure 6a). The synthesized signal is shown in Figure 6b. The amplitude of both sinusoidal signals is 0.5, and the resonance frequencies are 25 Hz and 86 Hz.

Even though the SNR is high at −6 dB, the maximum value of the protugram occurs at the central frequency of 300 Hz, which is far from the resonance frequency of 2500 Hz (see Figure 6c). The single peak value in the SES of the selected frequency band is near 61 Hz, which is the difference in the resonance frequency of the two sinusoidal signals (see Figure 6d). The PMgram is almost the same as that in Section 4.1. The optimal central frequency is 2483.22 Hz, near the resonance frequency of 2500 Hz (see Figure 6e), and the optimal bandwidth is 420 Hz. The SES of the optimal frequency band clearly indicates the existence of the impacts (see Figure 6f). In contrast, the optimal central frequency of the enhanced kurtogram is 312.5 Hz and the optimal bandwidth is 625 Hz at the fourth level (see Figure 6g). The single peak value in the SES of the optimal frequency band occurs at 61 Hz, which is the same as that of the protugram (see Figure 6h). It is clearly displayed in Figure 6i that the optimal central frequency and the bandwidth of the PMFSgram are 2616.1 Hz and 300 Hz, respectively. The optimal bandwidth is 1.5 times the repetitive frequency of the impacts. The SES of the optimal frequency band can also point out the existence of the impacts quite clearly (see Figure 6j). Both the protugram and the enhanced kurtogram are negatively affected by the single impulse in the SES or the power spectrum of the SE. However, the PMgram and the new method PMFSgram are not affected. Thus, the ability of the new method to resist the interference of a single impulse in the SES is proved.

## 5. Actual Vibration Data Tests and Recommendation

**Figure 7:**
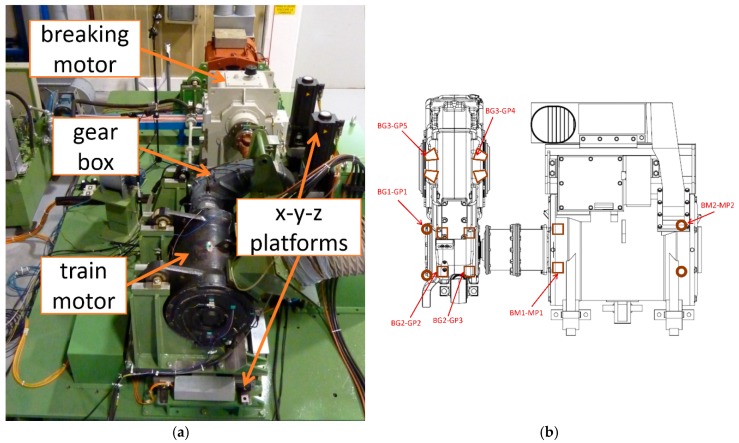
(**a**) The test-rig system; (**b**) The core schematic of the system.

**Figure 8:**
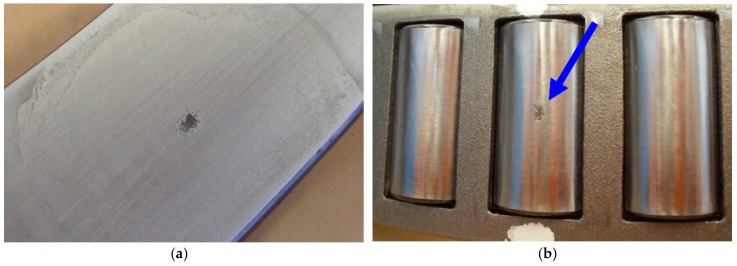
Two types of artificial defect on bearing: (**a**) spall on outer ring; (**b**) spall on roller

The performance of the PMFSgram is tested using actual vibration signals from rolling element bearings in a locomotive powertrain. The vibration signals have been obtained from a full-scale test-rig of the train traction system [29]. The train motor and gearbox are the same as those used on a real train. An image and a core schematic of the system are shown in Figure 7. The core of the system consists of the train motor and the gearbox (see Figure 7b). The x-y-z platforms are used to simulate the relative misalignment among train motor and gearbox shafts, due to track-induced vibrations. The tooth number of gears on the high-speed shaft and low-speed shaft are 26 and 85 respectively. The damaged bearing BG3-GP5 is a tapered roller bearing on the low speed shaft. Two types of bearing defect are tested with an artificial spall on the outer ring and the roller element respectively (see Figure 8). The parameters of the bearing are shown in Table 1.

The sampling frequency of both the vibration acceleration signals and the tachometer signals is 20,000 Hz. The length of the signals is 5 s with 10^5^ points. Cases of a constant speed and a slight acceleration of the speed are tested. The pre-processing is carried out to increase the SNR of the vibration signals and improve the performance of the new methods. According to Formulas (3) and (4), the fault frequencies BPFO and BSF are 18.4 NX and 7.43 NX in order domain. The total number of central frequencies for every bandwidth of the PMFSgram in the cases of outer ring defect is 30 and for the case of roller defect is 80. Because the fault order of roller defect is smaller so that it needs a smaller step size.

### 5.1. Experiment of Outer Ring Defect with a Constant Speed

**Figure 9:**
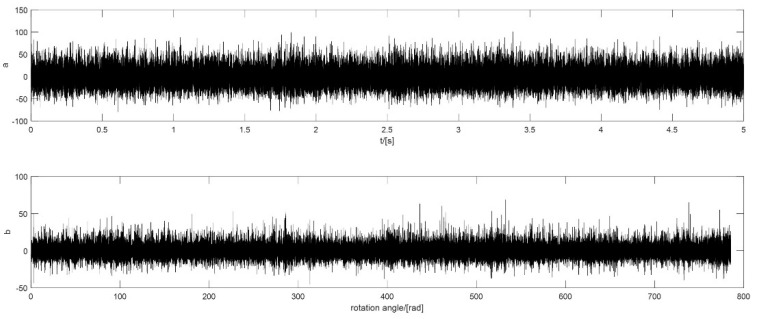
The vibration signal of outer ring defect with a constant speed: (**a**) the raw signal; (**b**) the signal after pre-processing.

In this case, the rotational speed of the motor is 5000 r/min, which is a relatively high speed. The defect is on the outer ring and the fault order is 18.4 NX. The raw signal and the signal after pre-processing are shown in Figure 9.

**Figure 10:**
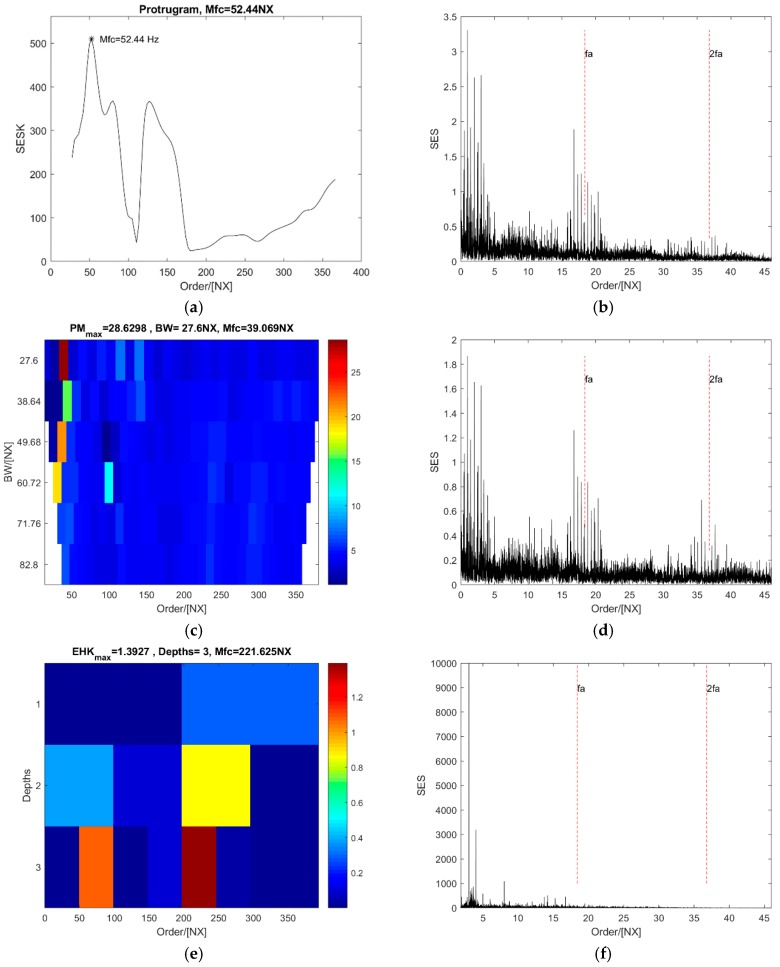

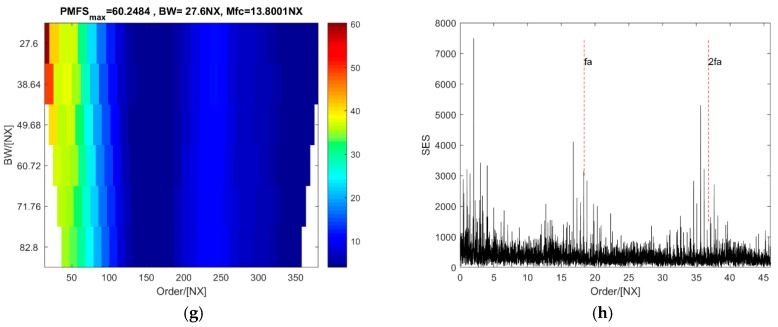
The analysis results of the case of outer ring defect with a constant speed: (**a**,**b**) the protugram and the SES of its optimal order band; (**c**,**d**) the PMgram and the SES of its optimal order band; (**e**,**f**) the enhanced kurtogram and the power spectrum of its optimal order band; (**g**,**h**) the PMFSgram and the SES of its optimal order band.

The optimal central order of the protugram is 52.44 NX (see Figure 10a), and the SES of the optimal order band can indicate the existence of a defect on the outer ring; however, the value at the fault order and its second harmonic are weak (see Figure 10b). Values at the fault order and its second harmonic are a bit stronger in the SES of the optimal order band of the PMgram than of the protugram (see Figure 10d). The optimal central order is 39.07 NX, and the optimal bandwidth is 27.6 NX for PMgram (see Figure 10c). Peak values at the first two fault order harmonics indicate the existence of defect on the outer ring. In contrast, the optimal central order in the enhanced kurtogram is 221.63 NX at the third level (see Figure 10e) and the optimal bandwidth is 49.6 NX. It is clearly shown in Figure 10f that the SES of the optimal order band has two peak points at 3 and 4 NX, while the value at the fault order and the second harmonic are almost zero that means no fault at the outer ring. However, in Figure 10h, two peak values obviously occur at the fault order and the second harmonic in the SES of the optimal order band selected by PMFSgram that clearly represents outer ring defect on the bearing. The optimal central order of the PMFSgram is 13.8 NX and the optimal bandwidth is 27.6 NX shown in Figure 10g. The optimal bandwidth is 1.5 times of the fault order. Therefore, it is verified that the new method is effective for actual vibration signals as well. The performance of the new method is the best of the four.

### 5.2. Experiment of Outer Ring Defect with a Slight Acceleration of the Speed

**Figure 11:**
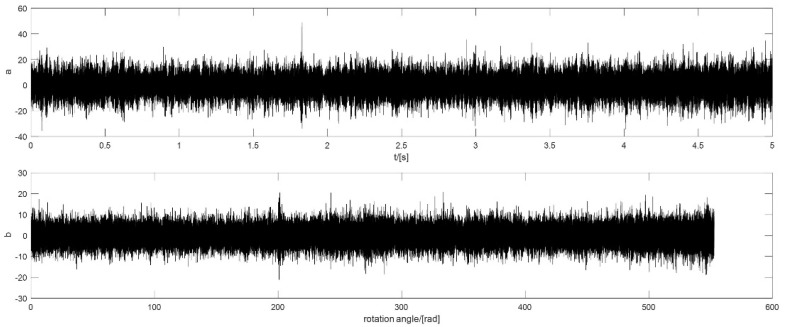
The vibration signal of outer ring defect with a slight acceleration of the speed: (**a**) the raw signal; (**b**) the signal after pre-processing.

In this case, the rotational speed of the motor increases from 3200 r/min to 5000 r/min in 5 min. The signal is a 5 s signal collected during the 5 mins. The defect is on the outer ring, because COT is carried out in the pre-processing stage so that the fault order is still 18.4 NX. The raw signal and the signal after pre-processing are shown in Figure 11.

**Figure 12:**
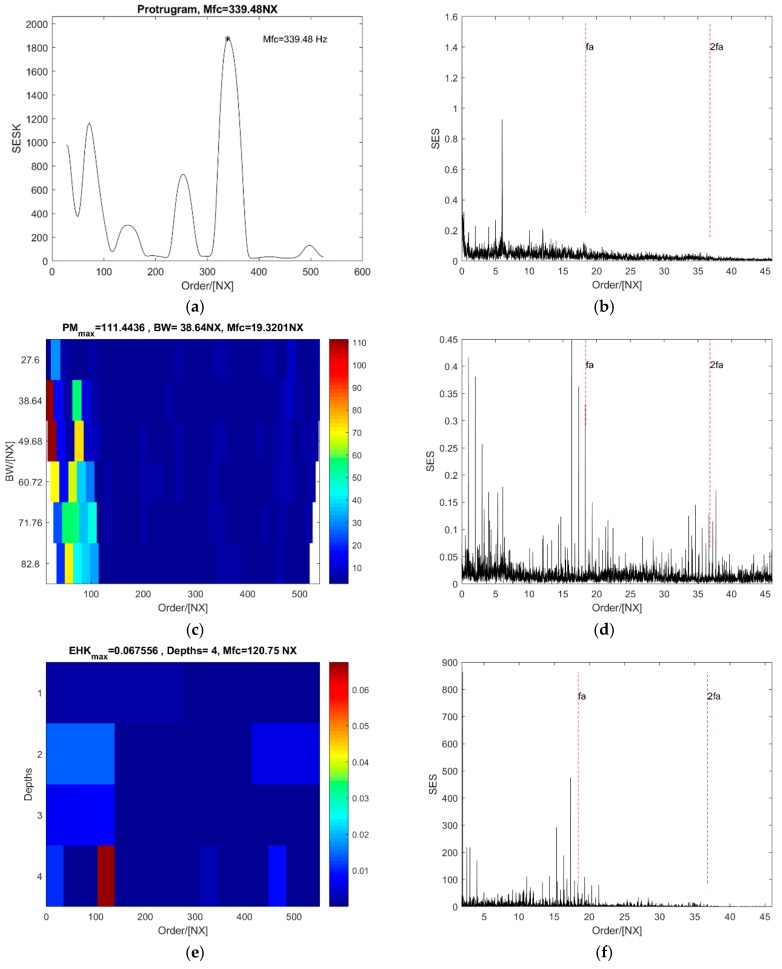

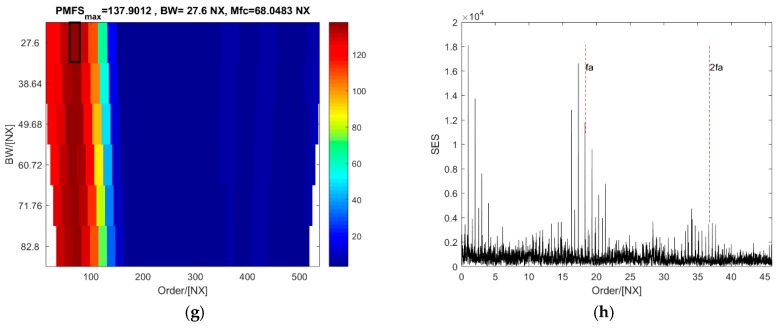
The analysis results of the case of outer ring defect with a slight acceleration: (**a**,**b**) the protugram and the SES of its optimal order band; (**c**,**d**) the PMgram and the SES of its optimal order band; (**e**,**f**) the enhanced kurtogram and the power spectrum of its optimal order band; (**g**,**h**) the PMFSgram and the SES of its optimal order band

The maximum value of the protugram occurs at order 339.48 NX (see Figure 12a). It is displayed in Figure 12b that the SES of the optimal order band has two peak values far from the fault order and values at the first two harmonics of fault order are masked in the noise. Compared with the SES of the optimal order band of the protugram, there are two peak values at the fault order and the second harmonic in the SES of the optimal order band of the PMgram (see Figure 12d). The optimal central order of the PMgram is 19.32 NX and the optimal bandwidth is 38.64 NX (see Figure 12c). In contrast, the optimal central order of the enhanced kurtogram is 120.75 NX and the optimal bandwidth is at the fourth level with the value of 34.5 NX (see Figure 12e). Even though the SES of the optimal order band can indicate the existence of a fault on the outer ring, the value at the fault order is quite weak (see Figure 12f), and a strong impulse occurs at order 2 NX. However, it is clearly shown in Figure 12h that there are two peak values at the fault order and the second harmonic, which indicates the existence of a fault on the outer ring apparently. The optimal central order of the PMFSgram is 68.05 NX and the optimal bandwidth is 27.6 NX displayed in Figure 12g. The optimal bandwidth is 1.5 times of the fault order. In this case, both protugram and the enhanced kurtogram select an optimal order band with few peak points in the SES of the optimal order band but are impossible to identify the defect. The performance of PMFSgram and PMgram are almost the same and superior to the other two.

### 5.3. Experiment of Roller Defect with a Constant Speed

**Figure 13:**
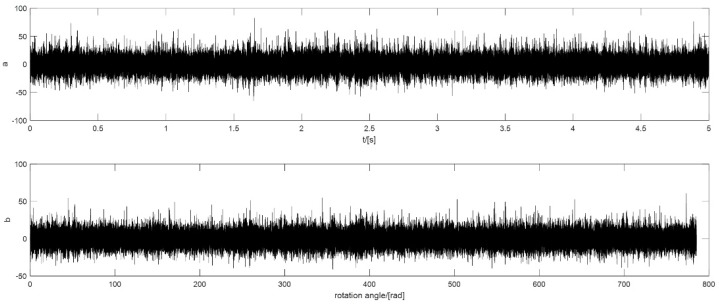
The vibration signal of roller defect with a constant speed: (**a**) the raw signal; (**b**) the signal after pre-processing.

In this case, the rotational speed of the motor is also 5000 r/min, but the defect is on one of the rollers, and the fault order is 7.43 NX. The raw signal and the signal after pre-processing are shown in Figure 13.

**Figure 14:**
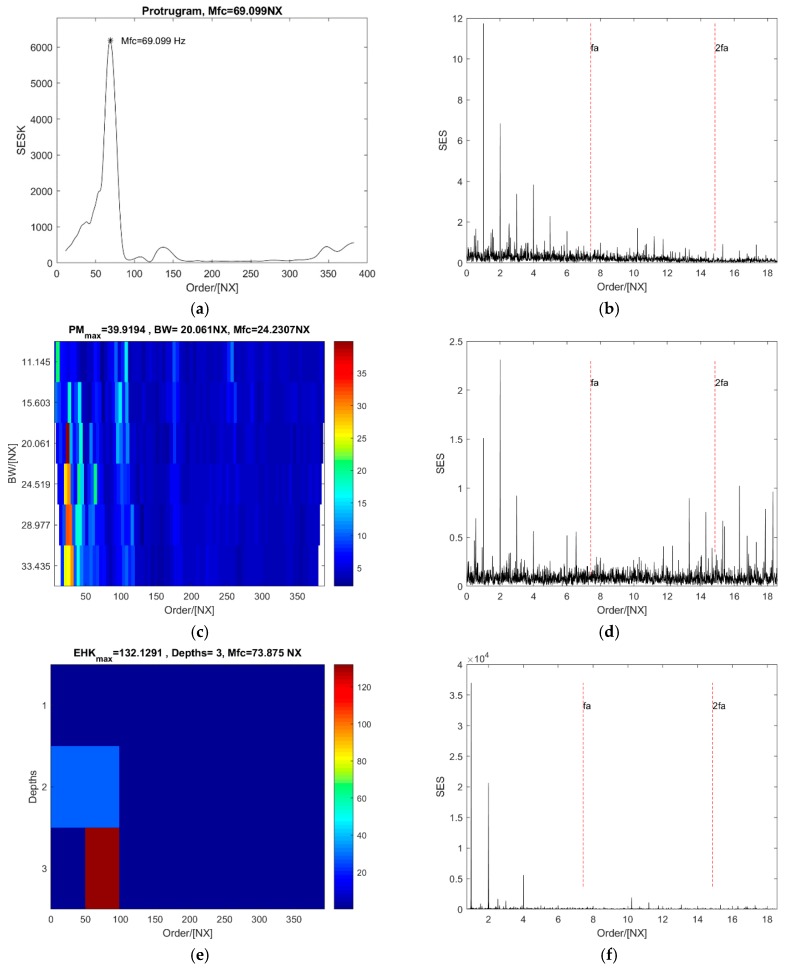

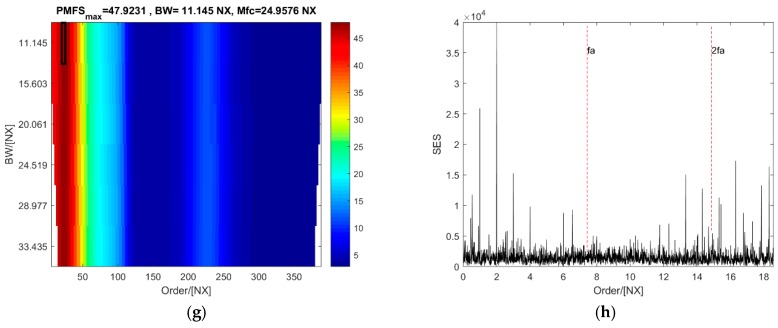
The analysis results of the case of roller defect with a constant speed: (**a**,**b**) the protugram and the SES of its optimal order band; (**c**,**d**) the PMgram and the SES of its optimal order band; (**e**,**f**) the enhanced kurtogram and the power spectrum of its optimal order band; (**g**,**h**) the PMFSgram and the SES of its optimal order band.

It is displayed in Figure 14a that the optimal central order of the protugram is 69.10 NX. It is difficult to show that it identifies the defect on the roller well, because the SES of the optimal order has only a very weak value at the second harmonic of the fault order (see Figure 14b). However, there is a clear peak value at the second harmonic of the fault order in the SES of the optimal order band of the PMgram (see Figure 14d). Furthermore, the optimal central order is 24.23 NX, and the optimal bandwidth is 20.06 NX. In contrast, it can be seen from Figure 14f that both values at the fault order and its second harmonic are close to 0 in the SES of the optimal order band of the enhanced kurtogram. But there are peak points at 1, 3, and 4 NX obviously. The optimal central order in the enhanced kurtogram is 73.88 NX and the optimal bandwidth is 49.6 NX at the third level (see Figure 14e). It is shown in Figure 14g that the optimal central order of the PMFSgram is 24.96 NX and the optimal bandwidth is 11.15 NX. The optimal bandwidth is 1.5 times of the fault order. The SES of the optimal order band of PMFSgram (Figure 14h) is almost the same as that of PMgram. Even though there is only a peak value at the second harmonic of the fault order, it still indicates there is a defect on the roller.

## 6. Conclusions

A new method in which just the envelope spectrum, in the range centred at the first two harmonics with a span of three times the modulation frequency, is used to calculate the kurtosis of the SES is proposed in this paper. It is quite different from the protugram and the enhanced kurtogram, which calculate the kurtosis on the whole SES. Both numerical experiments and actual vibration data tests demonstrated that the new method is effective in detecting an incipient local fault on an REB. The new method can overcome the common shortcomings of the protugram and the enhanced kurtogram, which are easily interfered with by a single or a few impacts in the SES or the power spectrum of the SE. Even though the performance of the PMgram is almost the same as that of the PMFSgram, but it is unnecessary to calculate a threshold for the PMFSgram. Thus, compared with the PMgram, the new method is easier to understand and operate. However, at present, the new method is just for detecting a single incipient fault on an REB. The application of this new method to cases in which multiple faults occur on a bearing at the same time needs to be researched further.

## Figures and Tables

**Table 1:** Parameters of bearing BG3-GP5.

Type	Inner Diameter	Outer Diameter	Roller Diameter ^1^	Pitch Diameter ^2^	Number of Rollers	Contact Angle
Value	236 mm	267 mm	17.37 mm	258.864 mm	39	12°

^1,2^ Roller diameter and pitch diameter are corresponding diameters passing the center of the defect.

## References

[1] Antoni J., Randall R.B. (2002). Differential diagnosis of gear and bearing faults. J. Vib. Acoust..

[2] Randall R.B., Antoni J., Chobsaard S. A comparison of cyclostationary and envelope analysis in the diagnostics of rolling element bearings. Proceedings of the IEEE International Conference on Acoustics, Speech, and Signal Processing.

[3] Ho D., Randall R.B. (2002). Optimisation of bearing diagnostic techniques using simulated and actual bearing fault signals. Mech. Syst. Signal Process..

[4] Abboud D., Antoni J., Sieg-Zieba S., Eltabach M. (2017). Envelope analysis of rotating machine vibrations in variable speed conditions: A comprehensive treatment. Mech. Syst. Signal Process..

[5] Antoni J., Randall R.B. (2006). The spectral kurtosis: Application to the vibratory surveillance and diagnostics of rotating machines. Mech. Syst. Signal Process..

[6] Antoni J. (2006). The spectral kurtosis: A useful tool for characterising non-stationary signals. Mech. Syst. Signal Process..

[7] Antoni J. The Spectral Kurtosis of nonstationary signals: Formalisation, some properties, and application. Proceedings of the 12th European Signal Processing Conference.

[8] Antoni J. (2007). Fast computation of the kurtogram for the detection of transient faults. Mech. Syst. Signal Process..

[9] Sawalhi N., Randall R.B., Endo H. (2007). The enhancement of fault detection and diagnosis in rolling element bearings using minimum entropy deconvolution combined with spectral kurtosis. Mech. Syst. Signal Process..

[10] Barszcz T., JabŁoński A. (2011). A novel method for the optimal band selection for vibration signal demodulation and comparison with the Kurtogram. Mech. Syst. Signal Process..

[11] Lei Y., Lin J., He Z., Zi Y. (2011). Application of an improved kurtogram method for fault diagnosis of rolling element bearings. Mech. Syst. Signal Process..

[12] Wang D., Peter W.T., Tsui K.L. (2013). An enhanced Kurtogram method for fault diagnosis of rolling element bearings. Mech. Syst. Signal Process..

[13] Chatterton S., Pennacchi P., Vania A., Borghesani P. A novel procedure for the selection of the frequency band in the envelope analysis for rolling element bearing diagnostics. Proceedings of the 9th IFToMM International Conference on Rotor Dynamics.

[14] Borghesani P., Pennacchi P., Chatterton S. (2014). The relationship between kurtosis-and envelope-based indexes for the diagnostic of rolling element bearings. Mech. Syst. Signal Process..

[15] Borghesani P., Pennacchi P., Ricci R., Chatterton S. (2013). Testing second order cyclostationarity in the squared envelope spectrum of non-white vibration signals. Mech. Syst. Signal Process..

[16] Antoni J. (2016). The infogram: Entropic evidence of the signature of repetitive transients. Mech. Syst. Signal Process..

[17] Lu S., He Q., Wang J. (2019). A review of stochastic resonance in rotating machine fault detection. Mech. Syst. Signal Process..

[18] Lu S., He Q., Zhang H., Kong F. (2017). Rotating machine fault diagnosis through enhanced stochastic resonance by full-wave signal construction. Mech. Syst. Signal Process..

[19] Song L., Wang H., Chen P. (2018). Vibration-Based Intelligent Fault Diagnosis for Roller Bearings in Low-Speed Rotating Machinery. IEEE Trans. Instrum Meas..

[20] Hao Y., Song L., Cui L., Wang H. (2018). A three-dimensional geometric features-based SCA algorithm for compound faults diagnosis. Measurement.

[21] Szabó L. Using maximum correlated kurtosis deconvolution method in the bearing fault detection of wind turbine generators. Proceedings of the 14th International Conference on Engineering of Modern Electric Systems (EMES).

[22] DeCarlo L.T. (1997). On the meaning and use of kurtosis. Psychol. Methods.

[23] Schoen R.R., Habetler T.G., Kamran F., Bartfield R.G. (1995). Motor bearing damage detection using stator current monitoring. IEEE Trans. Ind. Appl..

[24] Randall R.B., Sawalhi N., Coats M. (2011). A comparison of methods for separation of deterministic and random signals. Int. J. Cond. Monit..

[25] Fyfe K.R., Munck E.D.S. (1997). Analysis of computed order tracking. Mech. Syst. Signal Process..

[26] Braun S. (2011). The synchronous (time domain) average revisited. Mech. Syst. Signal Process..

[27] Borghesani P., Pennacchi P., Randall R.B., Sawalhi N., Ricci R. (2013). Application of cepstrum pre-whitening for the diagnosis of bearing faults under variable speed conditions. Mech. Syst. Signal Process..

[28] Antoni J., Randall R.B. (2003). A stochastic model for simulation and diagnostics of rolling element bearings with localized faults. J. Vib. Acoust..

[29] Pennacchi P., Bruni S., Chatterton S., Borghesani P., Ricci R., Marinis D., Didonato A., Unger-Weber F. A test rig for the condition-based maintenance application on the traction chain of very high-speed trains. Proceedings of the 9th World Congress on Railway Research.

